# Berberine Reduces Renal Cell Pyroptosis in Golden Hamsters with Diabetic Nephropathy through the Nrf2-NLRP3-Caspase-1-GSDMD Pathway

**DOI:** 10.1155/2021/5545193

**Published:** 2021-10-31

**Authors:** Baozhu Ding, Songyan Geng, Xiaojie Hou, Xuelian Ma, Huazhou Xu, Fan Yang, Kun Liu, Wenjie Liang, Guoping Ma

**Affiliations:** ^1^College of Integrated Traditional Chinese and Western Medicine, Hebei University of Chinese Medicine, Shijiazhuang 050091, Hebei, China; ^2^Hebei Key Laboratory of Integrative Medicine of Liver-Kidney Patterns, Institute of Integrative Medicine, Hebei University of Chinese Medicine, Shijiazhuang, Hebei, China; ^3^Department of Internal Medicine, Ningjin County Hospital, Xingtai 055550, Hebei, China; ^4^The First Hospital, Hebei Medical University, Shijiazhuang 050031, Hebei, China

## Abstract

**Objective:**

To observe the effect of berberine (BBR) on kidney cell pyroptosis in golden hamsters with diabetic nephropathy (DN) and to explore the molecular mechanism of its renal protection.

**Methods:**

Fifty clean-grade male golden hamsters were randomly divided into a control group (10) and a model building group (40). The DN model was established by high-sugar and high-fat feeding and injection of a small amount of STZ. After successful establishment of the model, they were randomly divided into a model group, western medicine group, and berberine high- and low-dose groups. The western medicine group was given irbesartan 13.5 mg/kg, and the berberine high- and low-dose groups were given BBR 200 mg/kg and 100 mg/kg, respectively, for 8 consecutive weeks. An automatic biochemical analyser was used to measure blood glucose, blood lipids, kidney function, MDA, and other indicators; radioimmunoassay was used to assess serum insulin; enzyme-linked immunosorbent assay (ELISA) was used to quantify IL-1*β*, IL-6, IL-18, TNF-*α*; HE, PAS, and Masson staining were used to observe kidney pathological tissue morphology; western blot and real-time fluorescent quantitative PCR were used to assess protein and mRNA expression of molecules, such as Nrf2, NLRP3, Caspase-1, and GSDMD; and TUNEL staining was used to detect DNA damage. SPSS statistical software was used for the data analysis.

**Results:**

The kidney tissues of golden hamsters in the control group were normal; Nrf2 was highly expressed, serum MDA level was low, NLRP3 expression in kidney tissue was not obvious, Caspase-1 and GSDMD were weakly expressed, and only a few TUNEL-positive cells were observed. Compared with the control group, the golden hamsters in the model group had obvious renal pathological damage; blood glucose, blood lipids, renal function-related indexes, insulin, and inflammatory factors IL-1*β*, IL-6, IL-18, and TNF-*α* were increased (*P* < 0.05); NLRP3, Caspase-1, and GSDMD expression was increased; Nrf2 expression was decreased; MDA level was increased (*P* < 0.05); and the number of TUNEL-positive cells was increased. Compared with the model group, the pathological morphology of the kidney tissue of golden hamsters in the three treatment groups was significantly improved; blood glucose, blood lipids, renal function, and the expression of inflammatory factors IL-1*β* and IL-6 were reduced (*P* < 0.05); NLRP3, Caspase-1, GSDMD, and other molecular proteins and mRNA expression were decreased; Nrf2 expression was increased; MDA level was decreased (*P* < 0.05); and the number of TUNEL-positive cells was decreased.

**Conclusion:**

DN golden hamster kidney NLRP3-Caspase-1-GSDMD signalling was enhanced. BBR can reduce oxidative stress damage by regulating antioxidative Nrf2 and then regulating NLRP3-Caspase-1-GSDMD signalling to inhibit pyroptosis, antagonizing DN inflammation-induced damage.

## 1. Introduction

Diabetic nephropathy (DN) accounts for 30% to 50% of end-stage renal disease (ESRD) cases and has become a public problem that seriously threatens the health of people worldwide [1]. An increasing number of studies have shown that [2] diabetes is characterized by chronic low-grade inflammation, also known as “metabolic inflammation.” DN is a diabetic complication caused by chronic inflammation. Inflammation plays a vital role in the occurrence and development of DN, but its exact mechanism has not yet been fully elucidated [3]. Pyroptosis is a recently discovered mode of programmed cell death accompanied by inflammation [4], a renal complication associated with hyperglycaemia, inflammation, and oxidative stress [5]. Oxidative stress leads to an increase in the level of reactive oxygen species (ROS), which affects various physiological and pathological processes, including inducing inflammation [6]. Nuclear factor erythroid-2-related factor 2 (Nrf2) is an important regulator of oxidative stress [7]. Nrf2 can regulate the expression of antioxidant protective genes and reduce systemic oxidative overload [8], thereby reducing oxidative stress and inflammation [9]. An important mechanism of inflammation is the induction of inflammasomes, which activate cysteinyl aspartate specific proteinase-1 (Caspase-1) and process inflammatory cytokines to cause pyroptosis. The NOD-like receptor protein 3 (NLRP3) inflammasome-mediated NLRP3-Caspase-1-GSDMD canonical pyroptosis pathway is an important mechanism of DN renal cell inflammatory damage [10]. Berberine (BBR) is an isoquinoline alkaloid mainly isolated from Coptis and Phellodendron. It has anti-inflammatory, antioxidant, hypoglycaemic, hypolipidaemic, and other pharmacological activities [11]. Previous studies have shown that BBR can delay the progression of DN by regulating related inflammatory factors and signalling pathways [12], but its exact mechanism has not been fully elucidated. Does BBR affect renal cell pyroptosis? Can BBR inhibit cells by regulating the oxidative stress regulator Nrf2? The pyroptosis pathway reduces inflammatory damage in DN kidneys, yet there are no reports on this mechanism. In this study, we observed the expression of Nrf2-NLRP3-Caspase-1-GSDMD pyroptosis pathway-related molecules in the kidneys of the DN golden hamster model and the regulation of BBR to explore its targets and molecular mechanisms, and we established a more similar type 2 diabetes model. The golden hamster model received BBR intervention, providing an experimental basis for clinical application and new drug development.

## 2. Materials and Methods

### 2.1. Animals

Clean-grade male golden hamsters were purchased from Beijing Weitong Lihua Laboratory Animal Technology Co., Ltd. (license number: SCXK (Beijing) 2016-0011). The experimental animals were housed in the Hebei Key Laboratory of Integrative Medicine of Liver-Kidney Patterns.

### 2.2. Drugs

Irbesartan (Ambovi) was purchased from Sanofi Pharmaceutical Co., Ltd.; berberine from Baoji Runyu Biotechnology Co., Ltd.; and high-sugar and high-fat feed from Nanjing Shengmin Scientific Research Animal Farm.

### 2.3. Reagents

The reagents used are as follows: Nrf2 antibody, Proteintech, lot number: 16396-1-AP; NLRP3 antibody, Abcam, lot number: ab263899; Caspase-1 antibody, Servicebio, lot number: GB11383; GSDMD antibody, Abcam, lot number: ab219800; *β*-actin antibody, Proteintech, lot number: 20536-1-AP; and TUNEL kit: Proteintech, lot number: PF00006.

### 2.4. Animal Grouping and Model Preparation

Fifty golden hamsters were randomly divided into a control group, model group, berberine high-dose group, berberine low-dose group, and western medicine group, with 10 hamsters in each group. After each group was fed normal feed for 1 week, the control group continued to be fed with normal feed, and the remaining 40 hamsters were fed a high-sugar and high-fat diet for 3 weeks. After the hamsters were fasted for 12 hours, STZ was injected intraperitoneally at 25 mg/kg for 3 consecutive days [13, 14], and the control group was intraperitoneally injected with citric acid buffer. Fasting blood glucose was detected 72 hours later, and the model was considered to have been successfully established at concentrations greater than 11.1 mmol/L. Then, the diabetic hamsters naturally developed DN.

### 2.5. Intervention Methods

After successful modelling, the berberine group was given BBR according to the literature [15], and the western medicine group was given irbesartan. The berberine high-dose group was administered 200 mg/kg, the berberine low-dose group was administered 100 mg/kg, and the western medicine group was administered 13.5 mg/kg. The control and model groups were given equal volumes of normal saline for 6 weeks of continuous intervention.

### 2.6. Index Detection and Methods

The general conditions of the hamsters were observed, including the number of deaths, mental state, activity, coat condition, food intake, drinking water, and urine output. After the intervention, urine was collected for 24 hours, and blood was taken from the groin. Some tissues were fixed in 4% paraformaldehyde for paraffin embedding, and the remaining tissues were stored at −80°C for index testing.

#### 2.6.1. Serum Index Detection

Blood was taken from the groin, 4 mL of the blood sample was saved, and serum was drawn. The following measures were detected: 24 h urine protein (24 h-UP); serum glucose (Glucose, GLU); body weight (Weight); kidney index (Kidney Index, KI), which is the ratio of kidney mass/weight; creatinine (Cr); uric acid (Uric Acid, UA); total cholesterol (TC); triglyceride (TG); low-density lipoprotein (LDL-C); insulin; malondialdehyde (MDA); the serum inflammatory factors interleukin-1*β* (IL-1*β*), interleukin-6 (IL-6), interleukin-18 (IL-18), and tumour necrosis factor-*α* (TNF -*α*); and other indicators.

#### 2.6.2. Staining of Kidney Pathological Sections

After routine fixation and paraffin embedding of all kidney tissues, the sections were deparaffinized with xylene, hydrated with gradient alcohol, and stained with haematoxylin and eosin (HE), periodic-acid Schiff (PAS), and Masson's trichrome stain.

#### 2.6.3. Real-Time PCR

The expression of NLRP3, Caspase-1, GSDMD, and IL-1*β* mRNA in kidney tissue was detected. RNA was extracted, and cDNA was reverse transcribed and then amplified. PCR was performed in a 20 *μ*L system under the following amplification conditions: 95°C predenaturation for 2 min; two-step cyclic reaction (95°C 10 s, 60°C 1 min) for 44 cycles; and melting curve for 95°C 15 s, 60°C 60 s, and 95°C 15 s. After amplification was completed, the Cq value of each target gene and the internal reference gene *β*-actin of each sample were obtained, the Cq value of the target gene−the Cq value of the internal reference gene *β*-actin = △Cq, according to formula Q = 2 − △Cq, the *Q* value and *Q* mean value of the target gene were obtained, and the *Q* value/*Q* mean value of each target gene, that is, the relative quantitative value (RQ value) of the expression of each target gene, was then used for statistical analysis. The primers were synthesized by the Shanghai headquarters of Shenggong, and the sequences are shown in Table 1.

#### 2.6.4. SABC Method

NLRP3, Caspase-1, and GSDMD protein expression was measured. Referring to the literature [16], the protein expression and position were observed under a microscope, and brown-yellow particles were observed under a light microscope as positive expression.

#### 2.6.5. Western Blot

The expression of Nrf2, NLRP3, Caspase-1, and GSDMD in kidney tissue was detected. Referring to the literature [17], the results obtained were corrected with an internal reference.

#### 2.6.6. TUNEL Staining

DNA damage in kidney tissue cells was detected. The kit instructions were followed for observation and analysis with a fluorescence microscope. CL488 is a green fluorescent dye with excitation wavelengths and emission wavelengths of 490 nm and 515 nm, respectively. Apoptotic cells were marked with bright green fluorescence.

### 2.7. Statistical Methods

SPSS 25.0 statistical software was used for data analysis. Data are expressed as $\bar{x}$ ± *S*, single-factor analysis of variance was used for multiple group comparisons, and the LSD test was used for comparisons between groups. *P* < 0.05 indicates a statistically significant difference.

## 3. Results

### 3.1. Comparison of General Conditions of Golden Hamsters in Each Group

Except for the hamsters in the control group, 40 golden hamsters were injected with a small amount of STZ; the modelling rate was 100%, 12 died, and the overall mortality was 30%. After intervention in each group, 2 animals died in the control group due to gavage, and 3 animals in each group died due to infection, diabetes complications, and other reasons in the model group and the three medication groups (Table 2).

During the experiment, the golden hamsters in the control group were generally in good condition, with normal diet, drinking water and urine output, good mental condition, and neat and shiny hair. The golden hamsters in the model group showed dull coats, increased food intake, sluggish movement, polydipsia, and polyuria. The above-mentioned symptoms of golden hamsters in the three treatment groups were improved to varying degrees compared with the model group.

### 3.2. Comparison of Blood Glucose and Blood Lipids of Golden Hamsters in Each Group

Compared with those in the control group, the GLU, TC, TG, and LDL-C levels of golden hamsters were increased in the model group (*P* < 0.05). Compared with those in the model group, the GLU, TC, TG, and LDL-C levels decreased in the three administration groups (*P* < 0.05) (Table 3).

### 3.3. Comparison of the Kidney Function of Golden Hamsters in Each Group

Compared with those in the control group, the 24 h-UP, Cr, and KI of the golden hamsters in the model group increased (*P* < 0.05). Compared with that in the model group, the expression of 24 h-UP, Cr, and KI decreased in the BBR high-dose group and the irbesartan group (*P* < 0.05); the 24 h-UP of the BBR low-dose group did not decrease significantly (*P* > 0.05) (Table 4).

### 3.4. Comparison of Serum Insulin in Each Group of Golden Hamsters

The serum insulin concentration of golden hamsters in the model group was significantly increased (*P* < 0.05), compared with that in the control group, the serum insulin concentration of the three administration groups decreased (*P* < 0.05), compared with that in the model group (Table 5).

### 3.5. Comparison of Serum MDA of Golden Hamsters in Each Group

The MDA concentration was significantly increased in the serum of golden hamsters in the model group (*P* < 0.05), compared with that in the control group; the MDA concentration in the three treatment groups decreased (*P* < 0.05), compared with that in the model group (Table 6).

### 3.6. Comparison of Serum Inflammatory Factor Levels in Golden Hamsters of Each Group

Compared with those in the control group, the serum levels of IL-1*β*, IL-6, IL-18, and TNF-*α* were significantly increased in the golden hamsters of the model group (*P* < 0.05). Compared with those of the model group, the serum IL-1*β* of the berberine high-dose group and the levels of IL-6, IL-18, and TNF-*α* were significantly reduced (*P* < 0.05). Serum IL-18 and TNF-*α* did not decrease significantly in the low-dose berberine group (*P* > 0.05) (Table 7).

### 3.7. Comparison of Pathological Morphology of Golden Hamster Kidneys in Each Group

#### 3.7.1. HE Staining

**Figure 1:**
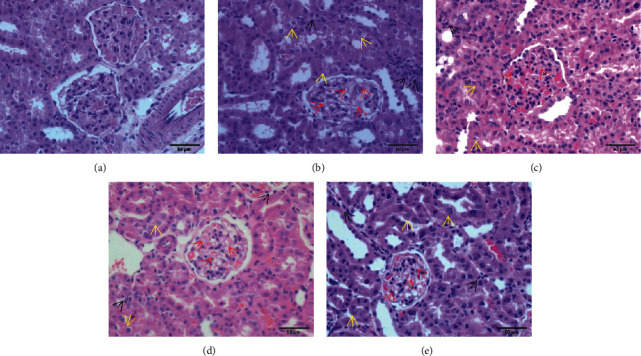
HE staining results of golden hamster kidney tissue in each group (×400). The red arrow in the figure mainly reflects the increase of mesangial matrix in the glomerulus; the yellow arrow reflects the degeneration and disorder of the cells in the renal tubules and the nuclear pyknosis of some tubular epithelial cells; the black arrow reflects the kidney infiltration of interstitial inflammatory cells. *Note.* (a) Control group; (b) model group; (c) BBR high-dose group; (d) BBR low-dose group; (e) irbesartan group.

No obvious pathological changes were observed in the structure or morphology of the golden hamster kidneys in the control group. Compared with those of the control group, the golden hamster glomeruli of the model group were clearly hypertrophic, the renal tubular epithelial cells were disordered and accompanied by vacuole-like degeneration, some of the renal tubules were atrophic, and the lumen of the renal tubules became narrow. Inflammatory cell infiltration can be seen in the renal interstitium. Compared with those in the model group, the pathological changes in the glomeruli and tubules in the kidney tissues of the three medication groups were significantly reduced (Figure 1).

#### 3.7.2. PAS Staining

**Figure 2:**
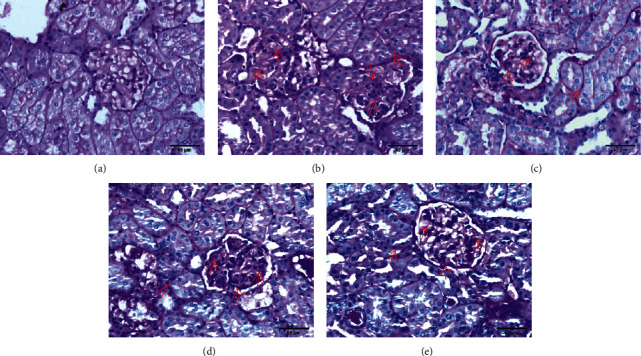
PAS staining results of golden hamster kidney tissue in each group (×400). The red arrow mainly indicates that the basement membrane is thickened, glycogen is increased, and PAS-positive deposits are increased. *Note.* (a) Control group; (b) model group; (c) BBR high-dose group; (d) BBR low-dose group; (e) irbesartan group.

In the control group, the glomerular capillary loops were thin and clear, and the kidney structure had no abnormal pathological changes. Compared with those of the control group, the glomerular capillary loops of the model group had a large amount of irregular and nodular PAS-positive deposits, the glomerular basement membrane was significantly thickened, the glomerular space was significantly narrowed, and some of the capillary loops had disappeared. Compared with those of the model group, the glomerular capillary loops of the three medication groups were clearer and more regular, there were fewer PAS-positive deposits, and glomerular space was visible (Figure 2).

#### 3.7.3. Masson Staining

**Figure 3:**
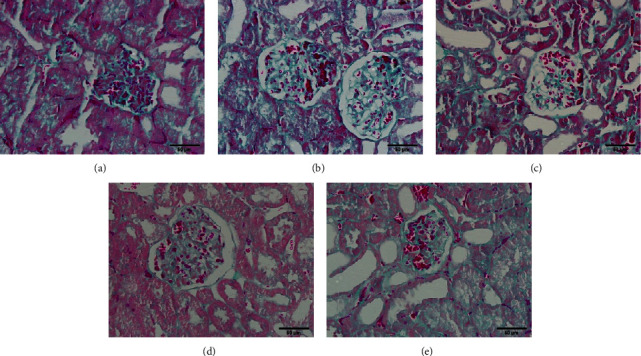
Masson staining results of the golden hamster kidney tissue in each group (×400). *Note.* (a) Control group; (b) model group; (c) BBR high-dose group; (d) BBR low-dose group; (e) irbesartan group.

Mesangial matrix hyperplasia and blue fibrous material deposition were rarely observed in the kidney tissue of the control group. Compared with that of the control group, the renal capsule in the kidney tissue of the model group was significantly enlarged, blue fibrous deposits were seen in the glomerulus, the glomerular mesangial area was significantly enlarged, and the mesangial matrix proliferated. Compared with those in the model group, mesangial matrix hyperplasia and blue fibre deposition were significantly reduced in the three drug groups (Figure 3).

### 3.8. Comparison of NLRP3, Caspase-1, GSDMD, and IL-1*β* mRNA Expression in the Kidney Tissue of Golden Hamsters in Each Group

Compared with that of the control group, the expression of NLRP3, Caspase-1, GSDMD, and IL-1*β* mRNA in the kidney tissue of the model group was increased (*P* < 0.05). Compared with that in the model group, the expression of NLRP3, Caspase-1, GSDMD, and IL-1*β* mRNA was decreased in the three medication groups (*P* < 0.05) (Table 8).

### 3.9. Comparison of NLRP3, Caspase-1, and GSDMD Protein Expression in the Golden Hamster Kidney Tissue of Each Group

#### 3.9.1. Immunohistochemical Results

**Figure 4:**
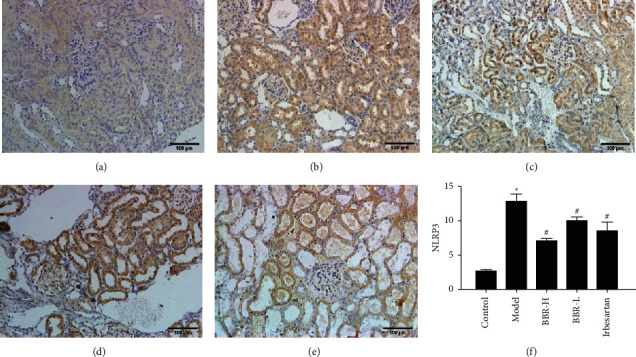
SABC detection of NLRP3 protein expression in golden hamster kidney tissue of each group (×200). *Note.* (a) Control group; (b) model group; (c) BBR high-dose group; (d) BBR low-dose group; (e) irbesartan group; (f) semiquantitative analysis of the positive expression of NLRP3.

**Figure 5:**
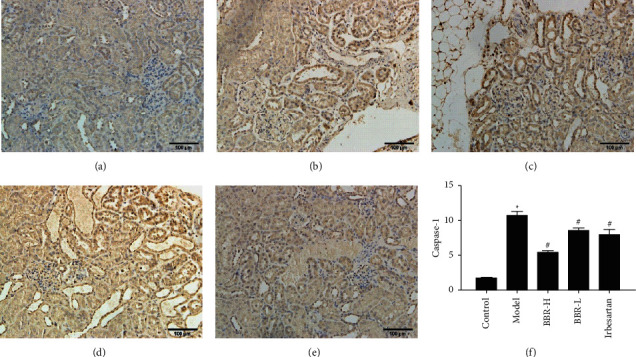
SABC detection of Caspase-1 protein expression in golden hamster kidney tissue of each group (×200). *Note.* (a) Control group; (b) model group; (c) BBR high-dose group; (d) BBR low-dose group; (e) irbesartan group; (f) semiquantitative analysis of the positive expression of Caspase-1.

**Figure 6:**
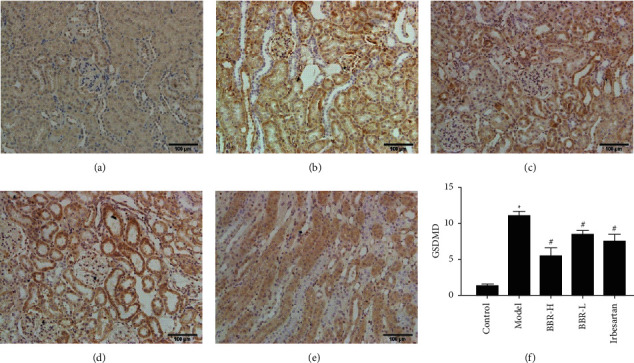
SABC detection of GSDMD protein expression in golden hamster kidney tissue of each group (×200). *Note.* (a) Control group; (b) model group; (c) BBR high-dose group; (d) BBR low-dose group; (e) Irbesartan group; (f) semiquantitative analysis of the positive expression of GSDMD.

NLRP3 in the model group was mainly expressed in renal tubular epithelial cells and glomeruli, and Caspase-1 and GSDMD were mainly found in renal tubular epithelial cells. The expression of NLRP3, Caspase-1, and GSDMD in the control group was weaker. Compared with that in the control group, the expression of NLRP3, Caspase-1, and GSDMD was increased in the model group. Compared with that in the model group, the protein expression of NLRP3, Caspase-1, and GSDMD in the three medication groups was decreased (Figures 4–6).

#### 3.9.2. Western Blot Results

**Figure 7:**
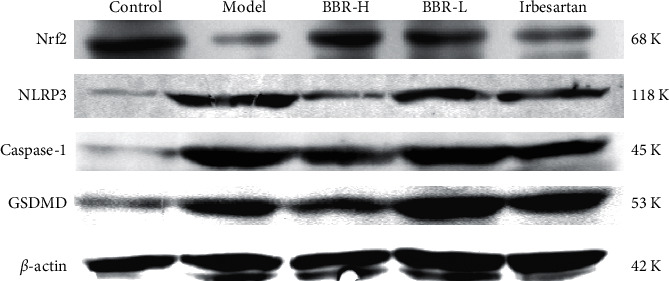
Western blot detection of Nrf2, NLRP3, Caspase-1, and GSDMD protein expression in golden hamster kidney tissues of each group (the uncropped images of all main blots have been provided as supplementary data (available here)).

**Figure 8:**
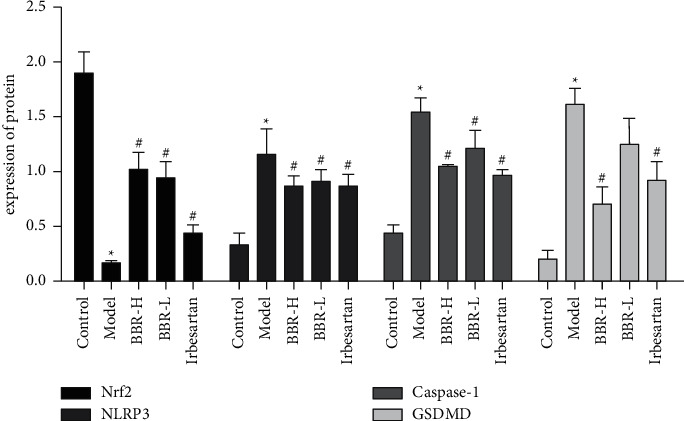
Comparison of Nrf2, NLRP3, Caspase-1, and GSDMD protein expression in golden hamster kidney tissues of each group.

In the control group, the expression of NLRP3, Caspase-1, and GSDMD was weak, and the expression of Nrf2 was strong. Compared with that in the control group, the protein expression of NLRP3, Caspase-1, and GSDMD was increased in the model group (*P* < 0.05), and the expression of Nrf2 decreased (*P* < 0.05). Compared with that in the model group, the expression of GSDMD protein was not significantly reduced in the BBR low-dose group (*P* > 0.05), and the expression of NLRP3, Caspase-1, and GSDMD protein decreased in the BBR high-dose group and irbesartan group (*P* < 0.05) (Figures 7 and 8; Table 9).

### 3.10. TUNEL Results

**Figure 9:**
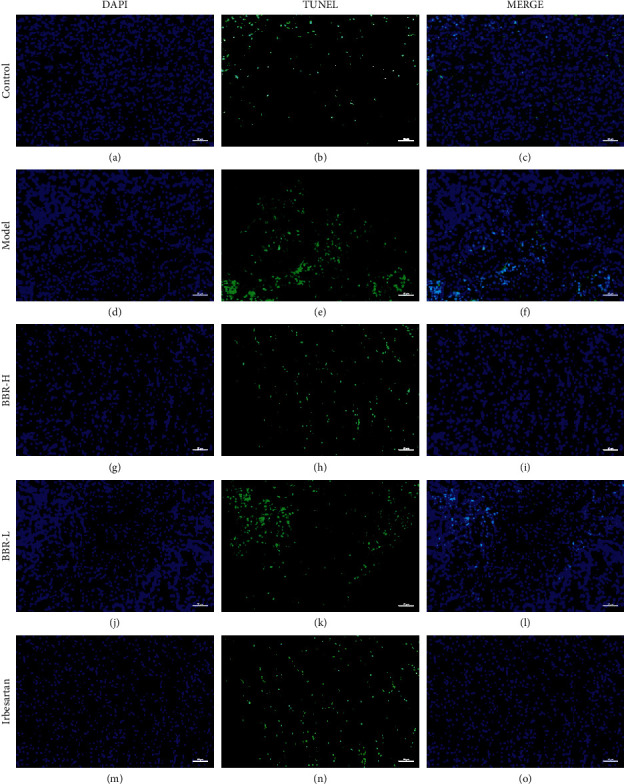
TUNEL staining results of golden hamster kidney tissue in each group. *Note.* In the TUNEL staining images, the blue DAPI is the nucleus; the green TUNEL is the DNA damage; and MERGE is the combination of the two (×200). (a–c) Control group; (d–f) model group; (g–i) BBR high‐dose group; (j–l) BBR low‐dose group; (m–o) irbesartan group.

**Figure 10:**
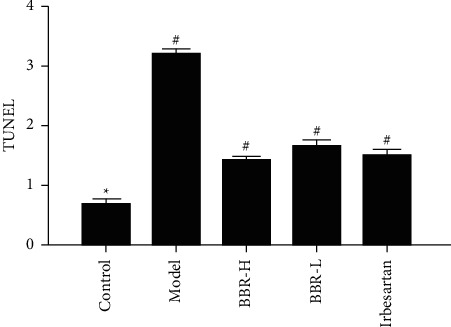
Quantitative analysis results of TUNEL staining of golden hamsters in each group.

Only a few TUNEL-positive cells were observed in the control group. Compared with that in the control group, the number of positive cells in the model group increased significantly, mainly the epithelial cells of the distal tubules with interstitial expansion. Compared with that in the model group, the number of TUNEL-positive cells in the three medication groups decreased (Figures 9 and 10).

## 4. Discussion

The pathogenesis of DN has not yet been fully clarified, but studies have shown that inflammatory damage is linked to the occurrence and development of DN [18]. Inflammatory cells, inflammatory factors, inflammasomes, and inflammatory pathways interact to eventually cause inflammatory damage. Pyroptosis is an inflammatory cell death method and an important mechanism of DN kidney inflammatory injury [19]. Tang et al. [20] reported that a high-fat diet and low-dose STZ can successfully induce a DN model in SD rats and confirmed that BBR can improve renal damage in DN rats. However, this study used golden hamsters, in which lipoprotein metabolism more closely resembles that in humans, and high-fat and high-sugar feeding combined with small doses and multiple consecutive STZ injections to successfully establish a diabetes model, effectively avoiding large doses that can easily cause animal death. At low doses, blood sugar will not be high. In addition, the increase in serum insulin levels in the model group reflects a certain degree of insulin resistance in golden hamsters, making the model more similar to type 2 diabetes [21, 22]. In this experiment, the expression of Nrf2-NLRP3-Caspase-1-GSDMD pyroptosis pathway molecules in the kidneys of DN golden hamsters was preliminarily studied, and the mechanism by which BBR improves inflammatory damage in DN kidneys was preliminarily explored.

BBR has a variety of pharmacological activities, such as anti-inflammatory, antioxidant, hypoglycaemic, and hypolipidaemic activities, and can be used for the treatment of type 2 diabetes, DN, hyperlipidaemia, heart disease, and other diseases [23]. Previous studies have found that BBR has a better protective effect on DN kidneys, including anti-inflammatory effects, regulation of metabolic abnormalities, antioxidative stress, and regulation of DN signal transduction pathways [24]. Pang et al. [25] reported that BBR can delay the progression of type 2 diabetes by regulating the body's inflammatory state. Jin et al. [26] showed that BBR has strong anti-inflammatory and antioxidant activities, which can activate AMP-dependent protein kinases and reduce the apoptosis of mouse podocytes induced by high glucose. Li et al. [27] found that BBR activates autophagy by inhibiting the mTOR/P70S6K/4EBP1 signalling pathway and reduces podocyte apoptosis. Huang et al. [28] reported that BBR improves renal function by reducing the expression and activity of SphK1 and S1P in the kidneys of diabetic mice induced by alloxan. This study used high- and low-dose groups to explore the regulatory effect of BBR on the Nrf2-NLRP3-Caspase-1-GSDMD signalling pathway to clarify the molecular mechanism by which BBR improves the renal function of DN.

### 4.1. Relieving Kidney Inflammation Damage Is an Important Mechanism by Which BBR Improves the Renal Function of DN

The main pathological features of DN caused by metabolic disorders include mesangial hyperplasia, glomerular hypertrophy, basement membrane thickening, and podocyte dysfunction [29]. Under diabetic conditions, BBR can reduce high blood sugar levels, regulate blood lipid metabolism disorders, and reduce kidney inflammation. Serum Cr and 24 h-UP are routine indicators that reflect renal function [30]. High- and low-dose BBR reduced serum Cr and 24-h UP levels, significantly reduced KI, and improved renal function. HE, PAS, and Masson staining revealed that DN golden hamsters showed glomerular hypertrophy, inflammatory cell infiltration, basement membrane thickening, renal tubular epithelial cell vacuolar degeneration, fibrous deposition, and other histopathological morphologies. BBR can therefore significantly reduce renal tissue pathology. IL-1*β*, IL-6, IL-18, and TNF-*α* are representative inflammatory factors, with IL-6 serving as a key component of the inflammatory mediator network. After the inflammatory response occurs, IL-6 is the first to be produced. IL-1*β* plays a central role in the inflammatory response and can promote the aggregation and adhesion of inflammatory cells and induce the inflammatory response. Similar to IL-1*β*, IL-18 needs to be cleaved by Caspase-1 of the inflammasome before it can be secreted to activate nuclear factor-kappa B (Nf-*κ*B), and NF-*κ*B then enters the nucleus to regulate the gene expression of inflammatory factors. TNF-*α* participates in local inflammation and endothelial cell activation and is the earliest and most important inflammatory cytokine produced by the body after being affected by pathogenic factors. High- and low-dose BBR significantly downregulated the expression of IL-1*β*, IL-6, IL-18, and TNF-*α* in DN kidney tissues and reduced renal inflammatory damage. In recent years, it has been found that BBR has a significant effect in the treatment of DN with dyslipidaemia and has been widely used in clinical treatment [31]. Lipid metabolism disorders in DN patients are often accompanied by severe atherosclerosis and vascular complications. Elevated TG and TC levels are salient features of DN patients. This study shows that BBR can significantly reduce serum TG, TC, and LDL levels and that inhibiting kidney inflammation may be the mechanism by which BBR improves lipid disorders [32].

### 4.2. Inhibition of Pyroptosis Is an Important Mechanism by Which BBR Reduces Inflammatory Damage in DN Kidneys

Pyroptosis is an inflammatory programmed cell death method that relies on caspases. It has the characteristics of apoptosis and necrosis and yet differs from them. DNA damage and membrane damage are two basic characteristics [33]. In the process of pyroptosis, various pathogen-associated molecular patterns (PAMPs) and damage-associated molecular patterns (DAMPs) trigger the formation of inflammasomes, thereby activating caspases, and activated caspase then cleaves gasdermin D (GSDMD), an effector protein of pyroptosis. After lysis, GSDMD combines with the lipids of the cell membrane to form membrane pores, and pyroptosis occurs [34]. Studies have confirmed that pyroptosis is related to oxidative stress. The reactive oxygen species produced by oxidative stress can activate the NLRP3 inflammasome, which in turn activates Caspase-1, promotes the production of IL-1*β* and IL-18, and leads to inflammation and pyroptosis [35]. While Nrf2 is involved in the regulation of oxidative stress, its expression is downregulated under high-glucose conditions, and its expression increases after medication. The results of this study showed that the expression of Nrf2 in the kidney tissue of DN golden hamsters in the model group was downregulated, and the levels of NLRP3, Caspase-1, and GSDMD mRNA and protein were all increased, suggesting that NLRP3-Caspase-1-GSDMD is the main renal pyroptosis pathway that aggravates kidney inflammation and damage. BBR can reduce oxidative stress by activating Nrf2 regulatory factors, inhibit the activation of NLRP3 inflammasomes, downregulate the expression of NLRP3 and Caspase-1 mRNA and protein in kidney tissue, significantly reduce the expression of the pyrolysis-effector protein GSDMD, reduce membrane damage, and reduce kidney organization of TUNEL-positive cells to reduce DNA damage.

### 4.3. Relieving Oxidative Stress Is an Important Mechanism by Which BBR Inhibits Pyroptosis

Experiments have demonstrated that both high and low doses of BBR can reduce insulin levels. Previous studies have shown [36] that oxidative stress can cause glucose metabolism disorders by damaging pancreatic islet B cells, which is an important mechanism for diabetes. Hyperglycaemia in patients with DN is due to insufficient insulin secretion in the patient's body or the body's resistance to insulin, and insulin-resistant tissue produces excessive inflammatory cytokines due to hypoxia. Therefore, inflammation and oxidative stress are closely related to the occurrence and development of DN. Heat-clearing medicines, such as Huanglian Shengdi, can protect pancreatic islet B cells, improve glucose metabolism, and reduce oxidative stress. This experiment shows that high- and low-dose BBR can improve the level of the oxidative stress indicator MDA and regulate insulin levels, thus playing an important role in the body's oxidation and antioxidant balance. Nrf2 is the main regulator of cell redox and a nuclear transcription factor. It can combine with the antioxidant response element (ARE) to protect downstream cells, and NQO1 transcription is activated, allowing it to prevent oxidative stress damage in the cell [37]. Studies have shown that [35] Nrf2 is involved in the regulation of oxidative stress, and its expression is downregulated under high-glucose conditions. Experiments have shown that both high and low doses of BBR can increase the expression of Nrf2 protein, indicating that the activation of Nrf2 may be a necessary condition for BBR to inhibit oxidative stress and regulate DN kidney cell pyroptosis.

Overall, DN golden hamster kidney NLRP3-Caspase-1-GSDMD signalling pathway molecule expression increased, and BBR could reduce oxidative stress damage by regulating Nrf2 antioxidant stress factor, reduce the generation of reactive oxygen species, and reduce the activation of NLRP3 inflammasomes. Then, the NLRP3-Caspase-1-GSDMD signalling pathway was regulated to inhibit DN golden hamster kidney cell pyroptosis and antagonize DN inflammatory damage, thereby improving renal function and slowing the progression of DN.

## Figures and Tables

**Table 1:** Real-time PCR primer sequences.

Gene	Upstream primers (5′ to 3′)	Downstream primers (5′ to 3′)	Length (bp)
NLRP3	CAGAGACTATGGTTGGGGCG	CTCCGATGTAGAGTCAAAGTTTCTG	196
Caspase-1	TGCAGATGCTTATGACAGCGA	CTATCTGACCAGCTTCGCCC	137
GSDMD	AAGTGCTGCGCAGTATTAGCA	TCTGGACCCACAAAGCCAAT	107
IL-1*β*	GCAGGCTCCGAGATGAACAA	TGTCCGTTGAGATGGAGAGC	79
*β*-Actin	TGAACGGGAAGCTCACTGGC	CATGTGAGATCCACGACGGACA	70

**Table 2:** Comparison of general conditions of golden hamsters in each group.

Group	Number of groups	Number of models	Modelling rate (%)	Number of deaths	Survival number	Mortality rate (%)
Control group	10	0	0	2	8	20
Model group	10	10	100	3	7	30
BBR-H group	10	10	100	3	7	30
BBR-L group	10	10	100	3	7	30
Irbesartan group	10	10	100	3	7	30

**Table 3:** Comparison of blood glucose and blood lipid of golden hamsters in each group ($\bar{x}$ ± *S*, mmol/L).

Group	*n*	GLU	TC	TG	LDL-C
Control group	8	9.3 ± 0.8	3.5 ± 0.3	2.8 ± 0.2	2.1 ± 0.3
Model group	7	35.6 ± 0.9^*∗*^	25.0 ± 2.7^*∗*^	27.0 ± 0.9^*∗*^	11.5 ± 1.0^*∗*^
BBR-H group	7	17.6 ± 0.8^#^	15.1 ± 0.6^#^	17.4 ± 0.8^#^	3.3 ± 0.4^#^
BBR-L group	7	23.9 ± 1.1^#^	18.5 ± 0.8^#^	20.9 ± 0.8^#^	6.4 ± 0.4^#^
Irbesartan group	7	28.6 ± 1.6^#^	22.2 ± 0.7	25.5 ± 0.8	5.4 ± 0.6^#^

*Note.* Compared with the control group, ^*∗*^*P* < 0.05; compared with the model group, ^#^*P* < 0.05.

**Table 4:** Comparison of kidney function of golden hamsters in each group ($\bar{x}$ ± *S*).

Group	*n*	24 h-UP (*μ*g/mL)	Cr (*μ*mol/L)	KI (%)
Control group	8	34.5 ± 6.8	16.9 ± 2.2	0.3 ± 0.04
Model group	7	84.3 ± 3.7^*∗*^	494.6 ± 32.6^*∗*^	1.4 ± 0.4^*∗*^
BBR-H group	7	58.6 ± 4.2^#^	217.7 ± 5.83^#^	0.5 ± 0.04^#^
BBR-L group	7	85.7 ± 7.4	337.9 ± 17.1^#^	0.7 ± 0.1^#^
Irbesartan group	7	25.8 ± 2.3^#^	144.8 ± 17.0^#^	0.5 ± 0.1^#^

*Note.* Compared with the control group, ^*∗*^*P* < 0.05; compared with the model group, ^#^*P* < 0.05.

**Table 5:** Comparison of insulin levels of golden hamsters in each group ($\bar{x}$ ± *S*).

Group	*n*	Insulin (mIU/L)
Control group	8	39.1 ± 10.8
Model group	7	137.6 ± 18.4^*∗*^
BBR-H group	7	51.4 ± 11.4^#^
BBR-L group	7	67.5 ± 11.1^#^
Irbesartan group	7	78.9 ± 4.7^#^

*Note.* Compared with the control group, ^*∗*^*P* < 0.05; compared with the model group, ^#^*P* < 0.05.

**Table 6:** Comparison of MDA of golden hamsters in each group (*x* ± *S*).

Group	*n*	MDA (*μ*mol/L)
Control group	8	11.2 ± 0.5
Model group	7	32.9 ± 1.6^*∗*^
BBR-H group	7	20.4 ± 1.4^#^
BBR-L group	7	24.8 ± 1.6^#^
Irbesartan group	7	17.0 ± 1.0^#^

*Note.* Compared with the control group, ^*∗*^*P* < 0.05; compared with the model group, ^#^*P* < 0.05.

**Table 7:** Comparison of serum inflammatory factors of golden hamsters in each group ($\bar{x}$ ± *S*, pg/mL).

Group	*n*	IL-1*β*	IL-6	IL-18	TNF-*α*
Control group	8	12.3 ± 2.2	15.9 ± 3.8	148.9 ± 20.2	15.7 ± 3.4
Model group	7	20.7 ± 4.7^*∗*^	24.4 ± 3.8^*∗*^	212.5 ± 21.9^*∗*^	22.6 ± 4.2^*∗*^
BBR-H group	7	15.9 ± 4.6^#^	17.7 ± 4.0^#^	167.1 ± 12.9^#^	17.2 ± 2.4^#^
BBR-L group	7	16.3 ± 0.2^#^	18.3 ± 0.8^#^	210.6 ± 31.7	20.3 ± 3.0
Irbesartan group	7	16.6 ± 1.4^#^	22.7 ± 3.1^#^	169.3 ± 6.4^#^	19.9 ± 1.4

*Note.* Compared with the control group ^*∗*^*P* < 0.05; compared with the model group ^#^*P* < 0.05.

**Table 8:** Comparison of NLRP3, Caspase-1, GSDMD, and IL-1*β* mRNA in the kidney tissues of golden hamsters in each group ($\bar{x}$ ± *S*).

Group	NLRP3	Caspase-1	GSDMD	IL-1*β*
Control group	0.34 ± 0.03	0.33 ± 0.18	0.05 ± 0.02	0.38 ± 0.04
Model group	1.66 ± 0.22^*∗*^	1.81 ± 0.04^*∗*^	2.51 ± 0.46^*∗*^	2.18 ± 1.45^∗^
BBR-H group	0.81 ± 0.16^#^	1.07 ± 0.07^#^	0.70 ± 0.24^#^	0.92 ± 0.33^#^
BBR-L group	1.00 ± 0.39^#^	1.27 ± 0.44^#^	1.22 ± 0.47^#^	1.39 ± 0.34^#^
Irbesartan group	0.57 ± 0.18^#^	0.81 ± 0.15^#^	0.73 ± 0.29^#^	0.33 ± 0.18^#^

*Note.* Compared with the control group, ^*∗*^*P* < 0.05; compared with the model group, ^#^*P* < 0.05.

**Table 9:** Western blot detection of Nrf2, NLRP3, Caspase-1, and GSDMD protein in golden hamster kidney tissues of each group ($\bar{x}$ ± *S*).

Group	Nrf2	NLRP3	Caspase-1	GSDMD
Control group	1.89 ± 0.19	0.11 ± 0.01	0.24 ± 0.09	0.30 ± 0.05
Model group	0.16 ± 0.03^*∗*^	1.23 ± 0.18^*∗*^	1.17 ± 0.05^*∗*^	1.01 ± 0.06^*∗*^
BBR-H group	1.01 ± 0.15^#^	0.58 ± 0.01^#^	0.69 ± 0.14^#^	0.52 ± 0.08^#^
BBR-L group	0.94 ± 0.15^#^	0.81 ± 0.12^#^	0.93 ± 0.10^#^	0.89 ± 0.09
Irbesartan group	0.43 ± 0.07^#^	0.75 ± 0.01^#^	0.82 ± 0.04^#^	0.75 ± 0.09^#^

*Note.* Compared with the control group, ^*∗*^*P* < 0.05; compared with the model group, ^#^*P* < 0.05.

## Data Availability

All data used to support the findings of this study are included within the article.

## Supplementary Materials

Supplementary Materials The uncropped images of all main blots.

## References

[1] An X., Zhang Y., Cao Y., Chen J., Qin H., Yang L. (2020). Punicalagin protects diabetic nephropathy by inhibiting pyroptosis based on TXNIP/NLRP3 pathway. *Nutrients*.

[2] Wang C., Hou X. X., Rui H. L. (2018). Artificially cultivated ophiocordyceps sinensis alleviates diabetic nephropathy and its podocyte injury via inhibiting P2X7R expression and NLRP3 inflammasome activation. *Journal of Diabetes Research*.

[3] Zhao W. C., Shen B. X., He C. Y., Jiang J. L. (2020). Mechanism of GSDMD-dependent pyroptosisin kidney damage in diabetic nephropathy. *Chinese Pharmacological Bulletin*.

[4] Shi J., Gao W., Shao F. (2017). Pyroptosis: gasdermin-mediated programmed necrotic cell death. *Trends in Biochemical Sciences*.

[5] Li F., Chen Y., Li Y., Huang M., Zhao W. (2020). Geniposide alleviates diabetic nephropathy of mice through AMPK/SIRT1/NF-*κ*B pathway. *European Journal of Pharmacology*.

[6] Wang S., Ji L. Y., Li L., Li J. M. (2019). Oxidative stress, autophagy and pyroptosis in the neovascularization of oxygeninduced retinopathy in mice. *Molecular Medicine Reports*.

[7] Kobayashi E. H., Suzuki T., Funayama R. (2016). Nrf2 suppresses macrophage inflammatory response by blocking proinflammatory cytokine transcription. *Nature Communications*.

[8] Donate-Correa J., Luis-Rodríguez D., Martín-Núñez E. (2020). Inflammatory targets in diabetic nephropathy. *Journal of Clinical Medicine*.

[9] Lazaro I., Lopez-Sanz L., Bernal S. (2018). Nrf2 activation provides atheroprotection in diabetic mice through concerted upregulation of antioxidant, anti-inflammatory, and autophagy mechanisms. *Frontiers in Pharmacology*.

[10] Fan Z. H., Ma F. F., Qu J., Li X. H. (2019). Research progress on the relationship between cell apoptosis and inflammation in diabetic nephropathy. *Journal of Chengdu Medical College*.

[11] Tang L. Q., Wang F. L., Zhu L. N., Lv F, Liu S, Zhang S. T (2013). Berberine ameliorates renal injury by regulating G proteins-AC- cAMP signaling in diabetic rats with nephropathy. *Molecular Biology Reports*.

[12] Wang Y.-Y., Tang L.-Q., Wei W. (2018). Berberine attenuates podocytes injury caused by exosomes derived from high glucose-induced mesangial cells through TGF*β*1-PI3K/AKT pathway. *European Journal of Pharmacology*.

[13] Jiang P., Sun Q. G., Xie P., Shi Y., Huang T. H. (2019). Protective effect of modified Jisheng Shengi decoction on kidney in rats with STZ-induced diabetic nephropathy. *Journal of Guangzhou University of Traditional Chinese Medicine*.

[14] He L., Gao S., Huang W., Liu G., Deng X. (2009). Diabetic golden hamsters fed with high-fat food induce kidney damage. *Chinese Journal of Pathophysiology*.

[15] Ni W. J., Ding H. H., Tang L. Q., Wei W. (2015). Effect of berberine on expression of vascular endothelial growth factor in diabetic nephropathy rats. *Chinese Pharmacological Bulletin*.

[16] Fang X., Tao D., Shen J., Wang Y., Dong X., Ji X. (2015). Neuroprotective effects and dynamic expressions of MMP9 and TIMP1 associated with atorvastatin pretreatment in ischemia-reperfusion rats. *Neuroscience Letters*.

[17] Ding W., Xu C., Wang B., Zhang M. (2015). Rotenone attenuates renal injury in aldosterone-infused rats by inhibiting oxidative stress, mitochondrial dysfunction, and inflammasome activation. *Medical Science Monitor*.

[18] Zhang J. P., Zhang H. H., Meng X. W. (2018). Effect of tangshenqing 2 on immune inflammatory molecules in diabetic nephropathy model mice. *Chinese Journal of Integrated Traditional and Western Medicine*.

[19] Shi J. (2020). Pyroptosis in metabolic diseases: recent progress. *Academic Journal of Second Military Medical University*.

[20] Tang L. Q., Liu S., Zhang S. T., Zhu L. N., Wang F. L. (2014). Berberine regulates the expression of E-prostanoid receptors in diabetic rats with nephropathy. *Molecular Biology Reports*.

[21] Kang Z., Chu X., Yang R., Ji M. (2014). Biomarkers of hyperlipidemia cholesterol metabolism in hamster. *Chinese Pharmacological Bulletin*.

[22] Qiao F. X., Shen Z. F., Ye F., Chen Y. T., Xie M. Z. (2000). Streptozotocin-diabetic hamster (an animal model to evaluate hypoglycemics and hypolipidemics). *Chinese Journal of Diabetes*.

[23] Yang G., Zhao Z., Zhang X. (2017). Effect of berberine on the renal tubular epithelial-to-mesenchymal transition by inhibition of the notch/snail pathway in diabetic nephropathy model KKAy mice. *Drug Design, Development and Therapy*.

[24] Ni W.-J., Ding H.-H., Tang L.-Q. (2015). Berberine as a promising anti-diabetic nephropathy drug: an analysis of its effects and mechanisms. *European Journal of Pharmacology*.

[25] Pang B., Zhao L. H., Zhou Q. (2015). Application of berberine on treating type 2 diabetes mellitus. *International Journal of Endocrinology*.

[26] Jin Y., Liu S., Ma Q., Xiao D., Chen L. (2017). Berberine enhances the AMPK activation and autophagy and mitigates high glucose-induced apoptosis of mouse podocytes. *European Journal of Pharmacology*.

[27] Li C., Guan X.-M., Wang R.-Y. (2020). Berberine mitigates high glucose-induced podocyte apoptosis by modulating autophagy via the mTOR/P70S6K/4EBP1 pathway. *Life Sciences*.

[28] Huang K., Liu W., Lan T. (2012). Berberine reduces fibronectin expression by suppressing the S1P-S1P2 receptor pathway in experimental diabetic nephropathy models. *PLoS One*.

[29] Ni W. J., Zhou H., Ding H. H., Tang L. Q. (2020). *Berberine ameliorates* renal impairment and inhibits podocyte dysfunction by targeting the phosphatidylinositol 3‐kinase-protein kinase B pathway in diabetic rats. *Journal of Diabetes Investigation*.

[30] Ma J., Rui H. P., Chen Q. Z., Wang Z. J., Chen Y. S. (2019). Study on anti⁃inflammatory and therapeutic properties of *Ganoderma lucidum* polysacch⁃arides on diabetic nephropathy in streptozotocin⁃induced mice. *Journal of Nan Jing Medical University*.

[31] Sun J., Chen X., Liu T. (2018). Berberine protects against palmitate-induced apoptosis in tubular epithelial cells by promoting fatty acid oxidation. *Medical Science Monitor*.

[32] Sun S.-F., Zhao T.-T., Zhang H.-J. (2015). Renoprotective effect of berberine on type 2 diabetic nephropathy in rats. *Clinical and Experimental Pharmacology and Physiology*.

[33] Yu Y., He L. J., Wang H. M. (2018). NLRP1 inflammasome promotes hyperglycemic and hyperinsulin-induced pyroptosis of glomerular mesangial cells. *Chinese Journal of Cellular and Molecular Immunology*.

[34] Martin-Sanchez D., Poveda J., Fontecha-Barriuso M. (2018). Targeting of regulated necrosis in kidney disease. *Nefrologia*.

[35] Zuo Y., Chen L., He X. (2021). Atorvastatin regulates MALAT1/miR-200c/NRF2 activity to protect against podocyte pyroptosis induced by high glucose. *Diabetes, Metabolic Syndrome and Obesity: Targets and Therapy*.

[36] Zheng Y., He X. (2021). Effect of heat-clearing herbs on metabolism and oxidative stress in type 2 diabetic mice and its mechanism. *Journal of Practical Traditional Chinese Internal Medicine*.

[37] Wu S., Wang Y. (2021). Effects of ginsenoside on the expression of Col IV, Nrf2 and NQO1 in rats with diabetic nephropathy. *Hebei Medical Journal*.

