# Corrigendum to “Berberine Reduces Renal Cell Pyroptosis in Golden Hamsters with Diabetic Nephropathy through the Nrf2-NLRP3-Caspase-1-GSDMD Pathway”

**DOI:** 10.1155/2022/9828973

**Published:** 2022-05-14

**Authors:** Baozhu Ding, Songyan Geng, Xiaojie Hou, Xuelian Ma, Huazhou Xu, Fan Yang, Kun Liu, Wenjie Liang, Guoping Ma

**Affiliations:** ^1^College of Integrated Traditional Chinese and Western Medicine, Hebei University of Chinese Medicine, Shijiazhuang 050091, Hebei, China; ^2^Hebei Key Laboratory of Integrative Medicine of Liver-Kidney Patterns, Institute of Integrative Medicine, Hebei University of Chinese Medicine, Shijiazhuang, Hebei, China; ^3^Department of Internal Medicine, Ningjin County Hospital, Xingtai 055550, Hebei, China; ^4^The First Hospital, Hebei Medical University, Shijiazhuang 050031, Hebei, China

In the article titled “Berberine Reduces Renal Cell Pyroptosis in Golden Hamsters with Diabetic Nephropathy through the Nrf2-NLRP3-Caspase-1-GSDMD Pathway” [1], there are some errors to be corrected as follows:

1. In Section 2.1, it should be stated that the animals were purchased from Hebei Yiweiwo Biological Technology Co., Ltd. (license number: SCXK (Hebei) 2018–002).
2. In Section 2.4, the description of how the animals were grouped should be clarified as follows: “Fifty golden hamsters were randomly divided into a control group (10) and a model building group (40). After successful establishment of the model, they were randomly divided into a model group, western medicine group, and berberine high and low-dose groups.”
3. In Section 3.1, “Comparison of General Conditions of Golden Hamsters in Each Group” should be clarified as follows: “Except for the hamsters in the control group, 40 golden hamsters were injected with a small amount of STZ, the successful model rate was 90% (36/40). After intervention in each group, 2 hamsters in the control group died by gavage and 1 failed to obtain materials, 8 hamsters in the experimental groups died due to infection, diabetes complications, and other reasons. The available remaining number in each group was 7 for data analysis”. The description of “3.1” is duplicated with the description of “Table 2.” The “3.1” description is more accurate.
4. For Tables 2–7, the sample size for the control group should be 7.
