# Supplementary figures and images for: Berberine Reduces Renal Cell Pyroptosis in Golden Hamsters with Diabetic Nephropathy through the Nrf2-NLRP3-Caspase-1-GSDMD Pathway
Source: Evid Based Complement Alternat Med. 2021 Oct 31;2021:5545193. doi: 10.1155/2021/5545193 (PMC9375700; doi:10.1155/2021/5545193)

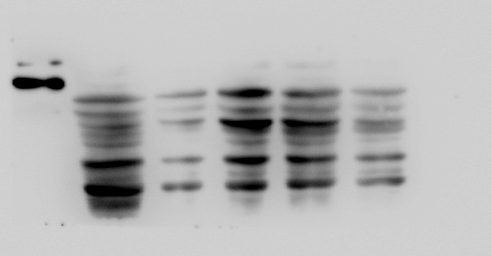

Supplement: Supplementary Materials — The uncropped images of all main blots. [file 5545193.f1.zip › 5545193.f1/1-Nrf2.jpg]

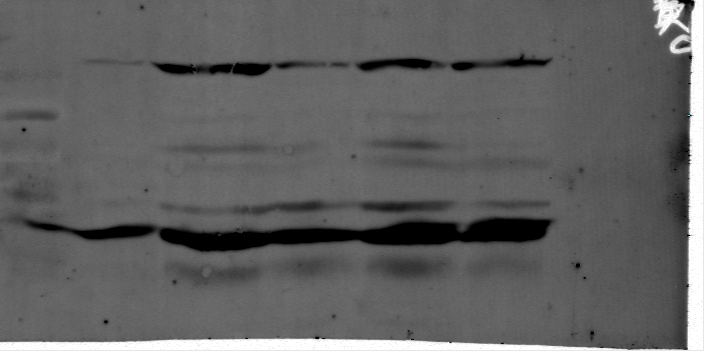

Supplement: Supplementary Materials — The uncropped images of all main blots. [file 5545193.f1.zip › 5545193.f1/2-NLRP3.jpg]

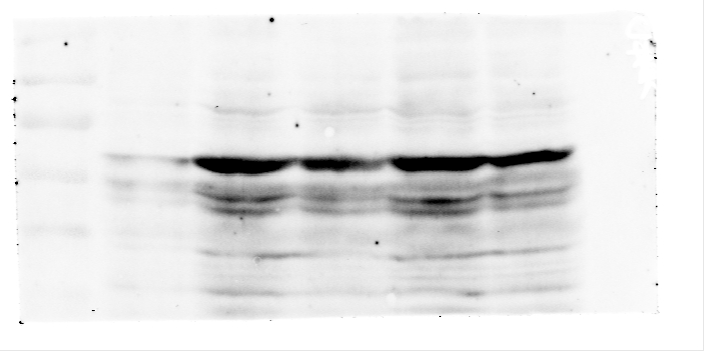

Supplement: Supplementary Materials — The uncropped images of all main blots. [file 5545193.f1.zip › 5545193.f1/3-Caspase-1.jpg]

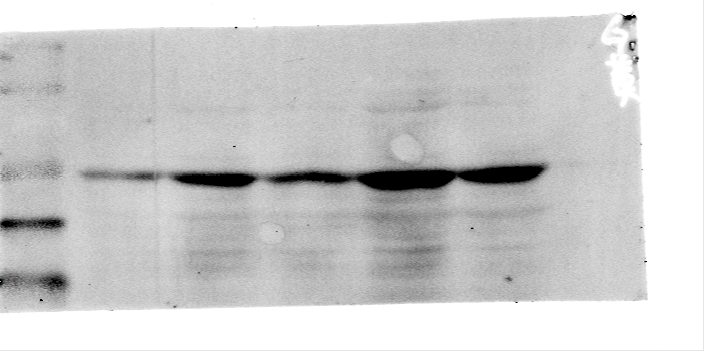

Supplement: Supplementary Materials — The uncropped images of all main blots. [file 5545193.f1.zip › 5545193.f1/4-GSDMD.jpg]
